# Real clinical experience after one year of treatment with tolvaptan in patients with autosomal dominant polycystic kidney disease

**DOI:** 10.3389/fmed.2022.987092

**Published:** 2022-09-29

**Authors:** Javier Naranjo, Francisco Borrego, José Luis Rocha, Mercedes Salgueira, Maria Adoración Martín-Gomez, Cristhian Orellana, Ana Morales, Fernando Vallejo, Pilar Hidalgo, Francisca Rodríguez, Remedios Garófano, Isabel González, Rafael Esteban, Mario Espinosa

**Affiliations:** ^1^Department of Nephrology, Hospital Universitario Puerta del Mar, Cádiz, Spain; ^2^Department of Nephrology, Complejo Hospitalario de Jaén, Jaén, Spain; ^3^Department of Nephrology, Hospital Universitario Virgen del Rocío, Seville, Spain; ^4^Department of Nephrology, Hospital Universitario Virgen del Macarena, Seville, Spain; ^5^Grupo de Estudio de la Enfermedad Poliquística Autosómica Dominante (GEEPAD), Granada, Spain; ^6^Department of Nephrology, Hospital de Poniente, El Ejido, Spain; ^7^Department of Nephrology, Hospital Universitario San Cecilio, Granada, Spain; ^8^Department of Nephrology, Hospital Universitario Puerto Real, Puerto Real, Spain; ^9^Department of Nephrology, Hospital Regional Universitario de Málaga, Málaga, Spain; ^10^Department of Nephrology, Hospital Costa del Sol, Marbella, Spain; ^11^Department of Nephrology, Hospital Universitario Torrecardenas, Almería, Spain; ^12^Department of Nephrology, Hospital Universitario Juan Ramón Jiménez, Huelva, Spain; ^13^Department of Nephrology, Hospital Universitario Virgen de las Nieves, Granada, Spain; ^14^Instituto de Investigación Biosanitaria ibs.GRANADA, Granada, Spain; ^15^Department of Nephrology, Hospital Universitario Reina Sofia, Córdoba, Spain

**Keywords:** glomerular filtration rate (eGFR), hepatic toxicity, polycystic kidney disease (PKD), tolvaptan, urinary osmolality

## Abstract

**Background:**

Tolvaptan (TV) is the first vasopressin-receptor antagonist approved for the treatment of autosomal dominant polycystic kidney disease (ADPKD). No publications report TV experience in real clinical practice during the first year of treatment.

**Methods:**

A prospective study of an initial cohort of 220 rapidly progressing patients treated with TV for 12 months. The tolerability of TV, the evolution of the estimated glomerular filtration rate (eGFR), analytical parameters, and blood pressure were analyzed.

**Results:**

A total of 163 patients (78.2%) received TV for 1 year. The main causes of treatment withdrawal were the aquaretic effects (11%), eGFR deterioration (5%), and hepatic toxicity (2.3%). eGFR decreased significantly after 1 month of treatment without further changes. The decrease in eGFR in the first month was higher in patients with an initially higher eGFR. The eGFR drop during the first year of treatment with TV was lower than that reported by patients in the 2 years prior to TV treatment (–1.7 ± 7.6 vs. –4.4 ± 4.8 mL/min, *p* = 0.003). Serum sodium and uric acid concentrations increased, and morning urinary osmolality decreased in the first month, with no further changes. Blood pressure decreased significantly without changes in antihypertensive medication.

**Conclusion:**

TV treatment is well tolerated by most patients. Liver toxicity is very rare and self-limited. TV reduces eGFR in the first month without showing further changes during the first year of treatment. Patients with a higher starting eGFR will suffer a greater initial drop, with a longer recovery. We suggest using the eGFR observed after a month of treatment as the reference for future comparisons and calculating the rate of eGFR decline in patients undergoing TV treatment.

## Introduction

Autosomal dominant polycystic kidney disease (ADPKD) is the fourth leading cause of end-stage renal disease in adults (1). It is mainly associated with mutations in the *PKD1* and *PKD2* genes (2). This mutation causes an alteration in the synthesis of polycystin-1 and two proteins and an accumulation of intracellular cyclic adenosine monophosphate (cAMP) that promotes the development and growth of renal cysts (3). Arginine-vasopressin (AVP) is an important inducer of intracellular cAMP production in the distal nephron mediated by its interaction with the vasopressin V_2_ receptor (4). Tolvaptan (TV), the first commercialized V_2_-receptor antagonist, has been shown to significantly reduce kidney volume growth and the rate of glomerular filtration (eGFR) decline, first in the TEMPO 3:4 trial and later in the REPRISE study with patients with more advanced kidney disease (5, 6). The main reported side effects of TV are those related to aquaresis (polyuria, frequency, and nocturia). In addition, more patients with increased liver enzymes were observed in the TV group. For this reason, when the European Medicines Agency approved the use of TV in ADPKD with evidence of rapid progression, it recommended monthly monitoring of liver enzymes during the first 18 months (7).

The published experience of TV treatment is very limited outside of clinical trials and probably comes from highly selected populations (8, 9). In this work, we analyze side effects and evolution during the first year of treatment with TV in ADPKD patients, in real clinical practice, in a large population in southern Spain, using different criteria of patient selection and different protocols of dose titration from those used in the aforementioned clinical trials.

## Materials and methods

**FIGURE 1:**
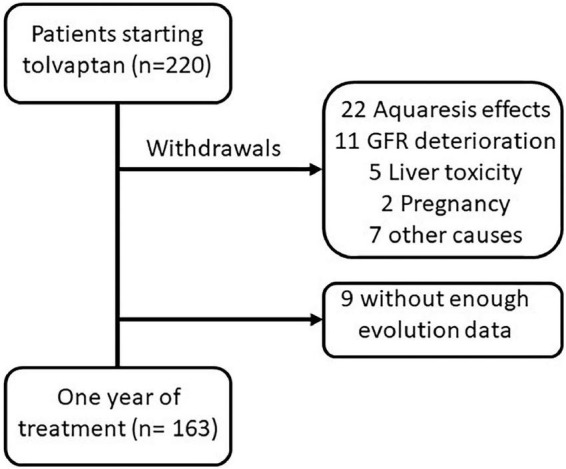
Flow chart of patients who started treatment with tolvaptan.

This is a prospective and multicenter study, with the participation of 14 hospital centers in Andalucía (Spain), including all patients who had started treatment with TV until October 2020. The date of the prescription for TV treatment was taken as the reference for each patient. Of a total of 220 patients who started treatment with TV during this period, 163 patients completed at least 1 year of treatment, which was the population of this study. The evolution of the patients who dropped out of treatment (not included in the study) up to that time was also reviewed. Reasons for withdrawal from TV treatment are shown in Figure 1. Hepatotoxicity was defined as an increase in transaminases or bilirubin of more than 3x the normal range. Aquaretic effects were defined as the presence of polyuria, frequency, and/or nocturia.

eGFR was estimated using the CKD-EPI equation (10). Given the early fall in eGFR observed at the start of TV treatment (11, 12), eGFR values presented in the first and third months were collected as the reference values for calculating the eGFR decline rate after 1 year. This will allow us to show the GFR decline caused by the progression of ADPKD itself, discounting the significant decline in GFR simply due to hemodynamic or volume depletion phenomena related to the effect of TV (12). The annual eGFR in the 2 years prior to TV treatment was also collected to calculate the rate of progression, expressing it on an annualized basis (mL/min/year).

Nephrologists prescribed TV treatment following their own criteria in each center in accordance with recommendations published by different models of considering a rapid progressor (RP) (13, 14). Total kidney volume (TKV) was measured by magnetic resonance imaging o computed tomography according to usual protocols in each center, but not in all patients, because of difficulties in performing these measurements or because of the usage of other models to define an RP (13). Patients with TKV measurements were classified by using adjusted TKV by height and age according to the Mayo Clinic classification (MCC) (15).

TV dose titration was performed according to the protocol established in each center. Liver enzymes and bilirubin were monitored monthly during the year of treatment. Since TV can cause some degree of volume depletion (11, 12), leading to a reduction in blood pressure, systolic and diastolic blood pressure measurements were also collected, along with antihypertensive medication taken at baseline and at 6 and 12 months. Morning urinary osmolality, serum sodium, and uric acid levels were collected at baseline, months 1, 3, 6, and 12. When considered, uric acid-lowering medications, allopurinol, and febuxostat were also reviewed.

Statistical analysis was performed with the r24-SPSS program. Results for categorical variables were presented with absolute and relative counts. Results for continuous quantitative variables were expressed as mean ± standard deviation. For comparing unpaired continuous quantitative variables, we used the Mann–Whitney test and the Wilcoxon test for paired samples. We used the McNemar and Pearson χ2 tests for unpaired samples to compare frequencies in paired samples. For the comparison of paired continuous quantitative variables in more than two samples, we used the Friedman test. For the correlation analysis, we used the Spearman coefficient. We considered significant values of *p* < 0.05.

## Results

The general characteristics of the 163 patients included in this study are shown in Table 1. eGFR decreased in patients with MCC 1C, 1D, and 1E from the first month with subsequent minor changes. No differences were found in the rate of eGFR decline when comparing MCC 1C, 1D, or 1E. Truncated or non-truncated *PKD1* mutations had a similar evolution of eGFR during the first year, with a comparable drop in the first month (Supplementary Table 1).

**TABLE 1:** Characteristics of study population before tolvaptan treatment.

	*n* = 164
**Gender, *n* (%)**	
Female	72 (44.2)
Male	91 (55.8)
**Age, years**	42 ± 8
**eGFR (mL/min/1.73 m^2^)**	62.8 ± 26.3
**CKD, *n* (%)**	
CKD1 (eGFR > 90 mL/min/1.73 m^2^)	22 (13.5)
CKD2 (eGFR 60–90 mL/min/1.73 m^2^)	52 (31.9)
CKD3a (eGFR 45–60 mL/min/1.73 m^2^)	50 (30.7)
CKD3b (eGFR 30–45 mL/min/1.73 m^2^)	34 (20.9)
CKD4 (eGFR 15–30 mL/min/1.73 m^2^)	5 (3.1)
**Mayo Clinic classification, *n* (%)** (*n* = 115; 70.6%)	52 (31.9)
1B	5 (4.3)
1C	23 (20.0)
1D	54 (47.0)
1E	33 (28.7)
**TKV (mL)**	2,329 (540–9,354)
**TKVh (mL/m)**	1,370 (333–5,920)
**Mutation, *n* (%)**	*n* = 100
Truncant *PKD1*	44 (44.0)
Non-truncant *PKD1*	47 (47.0)
*PKD2*	3 (3.0)
Not detected	6 (6.0)
**Systolic blood pressure (mmHg)**	132 ± 15
**Diastolic blood pressure (mmHg)**	83 ± 10
**Antihipertensive therapy (%)**	
ACEI/ARA-2	79.6
Calcium antagonists	22.8
α/β-blockers	18.5
Diuretics	13.0
**Albuminuria (mg/g)**	73 ± 126
**Morning urinary osmolality (mOsm/Kg)**	441 ± 156
**Serum sodium (mEq/L)**	140.7 ± 2.7
**Serum uric acid (mg/dL)**	6.1 ± 1.5

ACEI, angiotensin-converting enzyme inhibitors. ARA2, angiotensin-2 receptor antagonist. CKD, chronic kidney disease. eGFR, estimated glomerular filtration rate. PKD, polycystic kidney disease. TKV, total kidney volume. TKVh, total kidney volume adjusted by height.

The TV withdrawal rate was 21.8%. Most of the patients (45.8%) who discontinued treatment were due to side effects related to aquaresis. In five patients, elevated liver enzymes were detected with recovery to baseline values after the discontinuation of TV. In patients who discontinued treatment, the initial values and the evolution of the eGFR, serum sodium, uric acid, and morning urine osmolality were not significantly different from the values of the patients included in the study (Supplementary Table 2).

**FIGURE 2:**
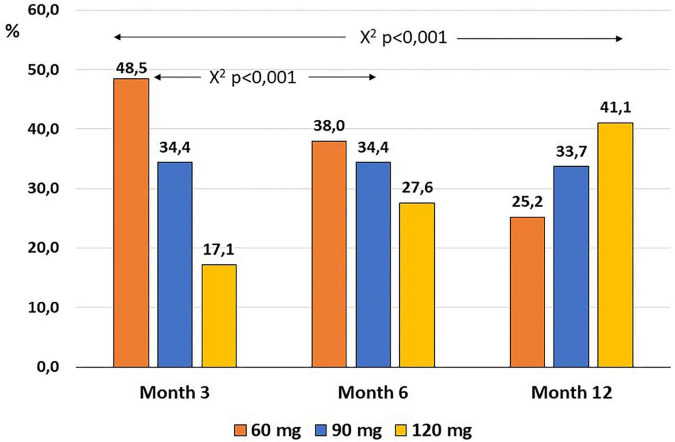
Evolution of the proportion of patients taking each dose of tolvaptan in the first year of follow-up.

TV dose at month 3 was 120 mg in 17.1% and 90 mg in 34.4% of the patients (Figure 2). The number of patients receiving 120 mg rose progressively to 41.1% at month 12, with a progressive decrease in patients receiving 60 mg to 25.2% (*p* < 0.001, Pearson’s χ2). For patients taking 60 mg of the TV at month 3, 30.8% increased the dose to 90 mg and 17.9–120 mg at month 12. For patients who were on 90 mg at month 3, 53.6% increased the dose to 120 mg, and the rest remained on 90 mg at month 12. We observe slight but not significant differences when comparing baseline mean eGFR values and the dose reached after 1 year of treatment (60 mg, 54.3 ± 17.6 mL/min/1.73 m^2^; 90 mg, 65.7 ± 26.9 mL/min/1.73 m^2^; 120 mg, 65.5 ± 29.4 mL/min/1.73 m^2^; Kruskal–Wallis *p* = 0.068).

### Evolution of the estimated glomerular filtration rate

eGFR decreased by 3.8 mL/min in the first month of treatment with TV, remaining stable during the first 6 months and decreasing to 5.9 mL/min on the 12th from baseline. When we compared the eGFR after 1 year of treatment with the eGFR after the first month of treatment with TV, we found that the mean total decline was only 2 mL/min (Table 2).

**TABLE 2:** Evolution of eGFR, globally and by subgroups of CKD, and for serum sodium, uric acid, and morning urinary osmolality during the first year of treatment with tolvaptan.

	Baseline	Month 1	Month 3	Month 6	Month 12
**eGFR (mL/min/1.73 m^2^)**	62.2 ± 26.4	58.3 ± 25.8^a^	58.9 ± 26.4^a^	58.5 ± 26.0^a^	56.8 ± 26.1^a^
Δ From baseline		–3.8 ± 8.3^a^	–3.7 ± 8.1^a^	–4.3 ± 8.2^a^	–5.9 ± 9.0^a^
Δ From the first month			0.03 ± 7.1	–0.4 ± 7.1	–2.0 ± 7.7^b^
**CKD stages**					
CKD1 (*n* = 22)	115.3 ± 21.4	106.6 ± 25.8^c^	109.4 ± 20.2^c^	107.5 ± 21.4^c^	104.8 ± 21.5^b^
Δ from baseline		–8.8 ± 13.8^c^	–5.9 ± 11.0^c^	–8.1 ± 13.2^c^	–11.1 ± 13.6^b^
Δ from the first month			2.9 ± 10.9	0.9 ± 10.6	–1.8 ± 10.0
CKD2 (*n* = 52)	70.6 ± 8.1	66.8 ± 10.8^b^	66.1 ± 13.3^b^	66.4 ± 11.7^b^	65.7 ± 13.8^a^
Δ from baseline		–3.9 ± 6.8^b^	–4.0 ± 8.8^b^	–3.9 ± 7.5^b^	–4.8 ± 9.4^a^
Δ from the first month			–0.6 ± 7.2	–0.2 ± 7.3	–1.1 ± 9.1
CKD3a (*n* = 50)	51.4 ± 4.9	47.5 ± 8.4^b^	47.5 ± 8.1^b^	47.6 ± 8.1^a^	45.5 ± 8.7^a^
Δ from baseline		–3.9 ± 7.0^b^	–3.9 ± 7.2^b^	–4.0 ± 7.0^a^	–6.1 ± 7.7^a^
Δ from the first month			0 ± 6.0	–0.1 ± 6.0	–2.1 ± 6.6
CKD3b (*n* = 34)	38.3 ± 4.7	37.3 ± 9.0	36.5 ± 8.6^c^	35.2 ± 8.6^c^	33.6 ± 8.0^a^
Δ from baseline		–1.1 ± 6.9	–1.9 ± 6.4^c^	–3.2 ± 6.4^c^	–4.8 ± 5.0^a^
Δ from the first month			–0.7 ± 5.5	–2.1 ± 5.9^c^	–3.7 ± 5.7^c^
CKD4 (*n* = 5)	23.2 ± 4.6	22.3 ± 5.4	22.6 ± 6.4	22.7 ± 6.3	21.6 ± 8.0
Δ from baseline		–0.9 ± 1.0	–0.6 ± 2.3	–0.5 ± 3.0	–1.6 ± 3.7
Δ from the first month			0.3 ± 2.0	0.4 ± 3.1	–0.7 ± 3.3
**Serum sodium (mEq/L)**	140.7 ± 2.7	141.8 ± 2.3^a^	141.6 ± 2.8^a^	141.4 ± 2.6^c^	141.7 ± 2.6^a^
**Serum uric acid (mg/dL)**	6.0 ± 1.5	6.5 ± 1.6^a^	6.5 ± 1.3^a^	6.4 ± 1.4^c^	6.4 ± 1.4^c^
**Morning urinary osmolality (mOsm/kg)**	454 ± 159	199 ± 67^a^	207 ± 92^a^	199 ± 73^a^	185 ± 52^a^

CKD, chronic kidney disease; eGFR, estimated glomerular filtration rate.

Δ = eGFR change from eGFR at baseline and from the first month.

^a^*p* < 0.001; ^b^*p* = 0.001; ^c^*p* < 0.05.

**FIGURE 3:**
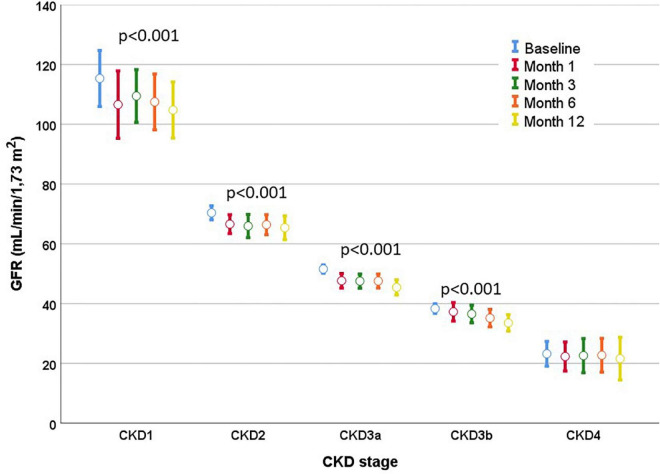
Evolution of the eGFR according to the stage of CKD. A drop in eGFR was observed in the first month, with subsequent stable values during the first year of treatment in all stages. Mean eGFR and mean standard errors are shown.

Attending the CKD stage, the eGFR decreased from the first month in CKD1 to CKD3a stages, while in the CKD3b stage, it was only significant from the third month, without observing any significant changes in the CKD4 stage (Figure 3 and Table 2). eGFR decreased more significantly in comparison to its baseline value in the CKD1 stage from the first month, with a final mean drop of –11.1 mL/min after 1 year of treatment compared to –4.8 to –6.1 mL/min in the CKD2 and CKD3b stages and only –1.6 mL/min in the CKD4 stage (Mann–Whitney test *p* = 0.037) (Supplementary Figure 1). However, the decline rates were more moderate when considering the eGFR in the first month as the reference for calculations for future declines; we found no significant differences in comparisons between CKD stages.

**FIGURE 4:**
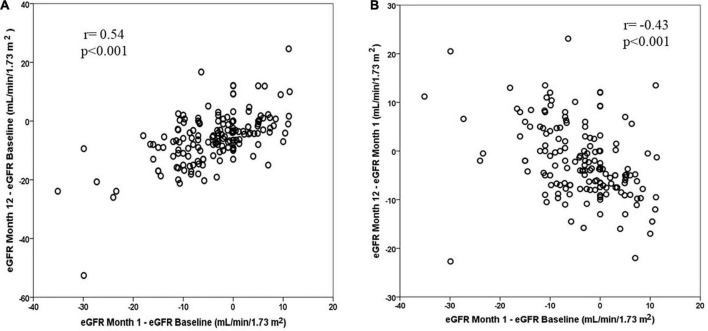
**(A)** Correlation between the change in eGFR in the first year from its baseline value and the change in eGFR in the first month from its baseline value. **(B)** Correlation between change in eGFR in the first year from the first month and the change in eGFR in the first month from the baseline value.

When the mean variation of eGFR in comparison to the first month was considered, patients with CKD1 stage showed a slight recovery of eGFR in the third month, while all showed little change between months 3 and 6 and at month 12 (Supplementary Figure 2 and Table 2). eGFR variation in the first month was positively correlated with the variation in the first year from baseline (*r* = 0.54, *p* < 0.001); that is, those who fell the most in eGFR in the first month were those who showed the greatest decrease after 1 year (Figure 4A). eGFR variation observed after 1 month of treatment was negatively correlated with the drop observed between month 1 and year 1 of treatment (*r* = –0.43, *p* < 0.001). Hence, patients in whom the eGFR fell the most in the first month subsequently experienced an improvement in eGFR between the first month and year 1 (Figure 4B). No relationship was found between the decrease in eGFR and the dose of TV that the patient was taking at each moment.

The mean rate of eGFR decline observed in the 2 years prior to starting TV treatment was –4.4 ± 4.8 mL/min/year (*n* = 128). However, in these patients, eGFR only decreased –1.7 ± 7.6 mL/min after 1 year of treatment without considering the first month (*p* = 0.003, Friedman’s test). Mean variations in eGFR were smaller in CKD2 to CKD4 stages compared to the previous 2 years (Supplementary Figure 3).

### Evolution of serum sodium and uric acid

**FIGURE 5:**
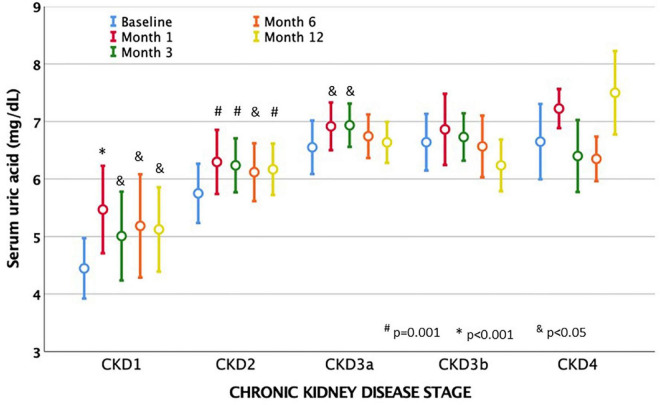
Evolution of mean serum acid uric levels after treatment with tolvaptan and chronic kidney disease stages (CKD). Acid uric levels increased from the first month of taking tolvaptan in all stages, but especially in patients con eGFR, which is more preserved. Baseline levels of uric acid gradually increased as eGFR dropped. Mean values and mean standard errors are shown.

Serum uric acid levels rose significantly from the first month and remained at higher levels compared to the start of treatment, with no significant differences when compared to months 1 or 3 (Table 2). The change observed in serum uric acid levels was weakly correlated with baseline eGFR (*r* = 0.16–0.21, *p* < 0.05), suggesting that patients with worse baseline eGFR had a smaller increase in their levels. Serum uric acid levels were lower before starting TV treatment in the early stages of CKD and increased more when eGFR was higher. The degree of increase in serum uric acid levels gradually decreased as eGFR decreased (Figure 5 and Supplementary Figure 4).

Before TV treatment, 19 patients took uric acid lowering drugs, while at months 3 and 6, the count rose to 30 patients and 36 patients at 1 year (McNemar’s test *p* = 0.007, *p* = 0.001, and *p* < 0.001, respectively). Given that serum uric acid levels are higher in men. With a lower eGFR, the prevalence of prescriptions for antihypertensive drugs was analyzed with a generalized linear repeated measures model, finding that only eGFR was a significant predictor of increased prescriptions. A progressive increase in the prescription for urate-lowering drugs from CKD stage 2 onward was detected (Supplementary Figure 5).

### Evolution of morning urinary osmolality

**FIGURE 6:**
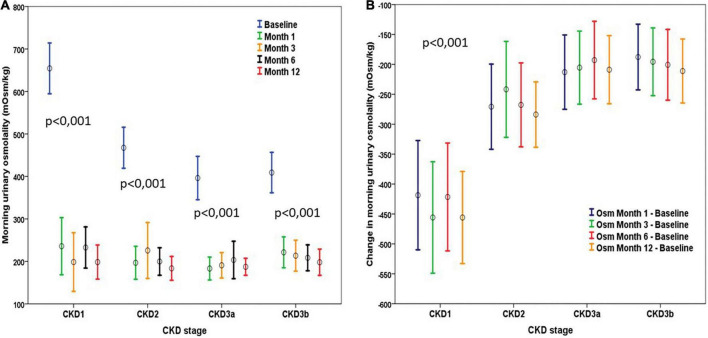
**(A)** Evolution of urinary osmolality according to the stage of CKD. There was a significant decrease in all stages from the first month, with no subsequent significant changes in any stage. **(B)** Change in urinary osmolality observed after tolvaptan according to CKD stage. Changes observed were stable during the first year of treatment within each class considered. The decrease in urinary osmolality was greater in the stages with a higher baseline GFR rate. Mean values and mean standard error are represented in both graphs.

Morning urinary osmolality decreased significantly from the first month of treatment after TV and remained later at similar values. When comparing urinary osmolality after 1 year in comparison to the first month of treatment, we observed a slight decrease (*p* < 0.001). Baseline morning urinary osmolality was correlated to baseline eGFR and dropped from the third month to remain at around 200 mOsm/kg, regardless of the CKD stage (Figure 6A). The degree of decrease in morning urinary osmolality was significantly higher in patients with higher baseline eGFR (Figure 6B). The decrease in urinary osmolality at each time was negatively correlated to baseline eGFR (*r* = –0.46 to –0.57, *p* < 0.01) and did not correlate with eGFR changes at that time or at future times.

### Evolution of blood pressure and antihypertensive medications

Systolic blood pressure decreased significantly from baseline to 6 months (132 ± 15 mm Hg vs. 125 ± 11 mm Hg, *p* < 0.001) and to 12 months (127 ± 12 mm Hg, *p* < 0.001), as well as diastolic blood pressure to 6 months (83 ± 10 mm Hg vs. 79 ± 10 mm Hg, *p* < 0.001) and to 12 months (78 ± 10 mm Hg, *p* < 0.001). We did not observe differences when comparing systolic or diastolic blood pressures according to the received dose of TV that they were receiving at 6 or 12 months. A reduction in the prescription for diuretics and an increase in consumption of α- or β-blockers were observed without changes in other antihypertensive groups (Supplementary Figure 6A). Without considering diuretics, we did not observe any change in the total number of antihypertensive drugs used during the first year (Supplementary Figure 6B).

## Discussion

The published experience with TV in ADPKD comes mostly from patients included mainly in two clinical trials (5, 6), with the same dose titration protocol and very homogeneous inclusion criteria. For this reason, we aimed to collect real clinical experience of the effects of TV in patients referred to a varied group of hospital centers that cover a broad region, such as Andalusia, in southern Spain, using different protocols for selecting patients and for the titration of TV doses.

Currently, the most accepted predictor of RP is the MCC (16, 17), as it has been shown to be the best predictor tool regardless of the CKD stage (17, 18). In our study, 95.6% of the included patients belonged to classes 1C to 1E of MCC. Some patients belonged to class 1B, aged > 48 years, but all had TKV > 750 mL and eGFR < 60 mL/min, criteria also used in the TEMPO 3:4 trial (5). Two young patients were included with TKV < 750 mL but belonged to classes 1C and 1D. Finally, the study included five patients aged > 55 years who were in accordance with the selection criteria of the REPRISE trial (6). Therefore, the population included in our study is probably more representative of patients visited in daily clinical practice, which is an added value of this study.

Titration of doses of TV was carried out in each center in a different way from that used in the TEMPO 3:4 study or included in the drug brochure. The number of patients on the higher doses increased over the months to finally reach 33.7% with 90 mg and 41.1% with 120 mg, compared to 21 and 55% in the TEMPO 3:4 trial (5). The decline in eGFR observed in the first year of treatment and the decrease in urine osmolarity were not related to the prescribed dose of TV. The withdrawal rate was 21.8%, caused mainly by aquaretic symptoms, similar to what has already been published (5, 6). The increase of liver enzymes or bilirubin improved after the discontinuation of TV, and no relationship was observed with the taken dose. We did not find more side effects in patients with a higher dose of TV. From our experience, TV is a safe drug that is acceptably tolerated by the majority of patients.

In our study, we observed the well-known early reduction of eGFR demonstrated in the first month of treatment (11, 19), which is attributed to hemodynamic phenomena and changes in tubuloglomerular feedback (12). In these pilot studies, eGFR fell in the first weeks by 3–8.6% with a reduction in filtration fraction and without clear changes in renal plasma flow (11, 12, 19). This reduction in GFR occurred in patients with higher initial GFR, which is accompanied by higher urine output and, therefore, greater chances of suffering volume depletion and a reduction of eGFR (19). We observed a particularly important eGFR drop in the CKD1 stage, which may cause concern in nephrologists who prescribe TV. However, we observed a clear recovery of eGFR at month 3 in this group, which indicates an initial volume depletion and hypothetical hemodynamic or tubuloglomerular feedback changes of reversible nature that should not cause concern or treatment withdrawal. In addition, a rise in eGFR is frequently observed when TV is suspended, even in patients with advanced CKD (6, 20).

In our study, we found a decline in eGFR at the end of the first year of around –6 mL/min/year, a rate much higher than –2.7 mL/min/year in the TV group or –3.7 mL/min/year in the placebo group in the TEMPO 3:4 study; –2.34 mL/min/year in the TV group and –3.61 mL/min/year in the placebo group in the REPRISE trial (5, 6). This difference was especially important considering the drop in CKD1 stage: –11.1 mL/min/year compared to the baseline value. However, when the slope of eGFR deterioration was calculated in comparison to the first month, the drop was –2 mL/min/year overall and only –1.8 mL/min/year in the CKD1 stage, which is in agreement with the results of the TEMPO 2:4 study in the first year (21). In the REPRISE trial, calculation of the GFR decline slope was performed considering values before and after the discontinuation of TV treatment, so this initial drop in GFR was not included in the GFR decline slope (6). In the TEMPO 3:4 trial, the calculation of changes in the slope of the inverse of creatinine was made by comparing its value always while taking TV, at the beginning of the trial after having carried out the dose escalation and, in the last review, before suspending TV, thus also without taking into consideration this initial GFR drop (5). Therefore, when calculating the rate of eGFR decline during TV treatment, we propose using the eGFR present in the first month of treatment as a starting point for future comparisons, thus avoiding exaggerated estimations for changes in GFR.

Serum sodium and uric acid levels increased significantly, as has been widely described (5, 6, 11, 12). Natremia and uricemia rose significantly in the first month but without clinical repercussions and subsequently remained in a similar range. The rise in uric acid can be observed early with a degree of increase that is greater than the fall in eGFR and is not related to a decrease in renal blood flow (2, 8). We observed a greater rise in serum uric acid levels in patients with higher baseline eGFR, which can be explained by higher urine output and greater proximal reabsorption of sodium to compensate for the reduction in distal sodium reabsorption caused by V_2_-receptor blockade (19). Accompanying this rise in serum uric acid, we found an increase in the prescription for allopurinol and febuxostat during the follow-up years.

After starting TV, urinary osmolality fell clearly and remained stable after the third month of treatment. The amount of reduction was clearly related to baseline eGFR, as already described, being higher in patients with higher eGFR (19, 22). In the TEMPO 3:4 trial, a greater reduction in urinary osmolality was proposed as a predictive marker of less future eGFR deterioration (22). However, this statement was based only on a stratified analysis of the CKD stage, which was inconclusive. Morning urinary osmolality was higher in patients with higher eGFR at baseline, who were also the ones to show the least reduction in eGFR at the end of the follow-up of the trial. We have not observed any relationship between eGFR decline and baseline urinary osmolality. We did not observe a greater decrease in urinary osmolality when comparing the different doses of TV.

A *post hoc* analysis of the TEMPO 3:4 study showed a slight decrease in systolic and diastolic blood pressure, which could not be attributed to changes in medication. In some cases, this was accompanied by a significant reduction in body weight, which would indicate body volume depletion after TV treatment, at least initially (23). We have also found a decrease in systolic and diastolic blood pressure without changes in the total antihypertensive count during follow-up.

Our study has some limitations despite the significant number of patients included. One year of duration is not enough to analyze long-term eGFR evolution. Our main aim was to show the evolution in the short term. However, a very interesting contribution that we make is to show how to calculate the eGFR decline rate by discounting the initial drop, which will be useful in daily practice for future observations. The heterogeneity in the selection of patients in our study should be seen as a strength since it is closer to real clinical practice. Our data reproduce the published results with TV despite using a slower TV dose titration than that used in the TEMPO 3:4 trial, but also consistent with the recommendations of some authors (24). It would have been interesting to analyze the eGFR evolution in each class of the Mayo classification, but the limited number of patients available prevented it.

## Conclusion

In conclusion, TV is a drug with good tolerability in most patients and a low rate of side effects. TV causes a reduction in eGFR in the first month of treatment with subsequent maintenance of values during the first year of follow-up. The fall in eGFR during the first year seems to be less than that reported by the patients prior to starting treatment with TV. Morning urinary osmolality and eGFR decreased more in patients with higher initial eGFR, remaining unchanged throughout treatment. We suggest calculating the rate of eGFR decline in patients undergoing TV treatment, using eGFR the first-month eGFR as a baseline value, in order to discount the initial fall due simply to functional changes in eGFR.

## Data availability statement

The original contributions presented in this study are included in the article/Supplementary material, further inquiries can be directed to the corresponding author/s.

## Author contributions

JN and FB contributed to the writing of the manuscript. JN collected all the data. FB performed the statistical analysis. All authors provided patient data, contributed to the review of the manuscript, read and approved the submitted version.

## Supplementary material

Supplementary Figure 1 Changes in eGFR compared to its baseline value, according to the stage of CKD, during the first year of treatment with tolvaptan. A progressive decrease in eGFR was observed from the first month of treatment in all stages except stage CKD4. The most significant decrease was in the CKD1 stage. Mean values and mean standard errors are shown.

Supplementary Figure 2 Mean difference of the eGFR compared to its value in the first month of treatment with tolvaptan. Changes observed were not significant in any stage of CKD. For comparison purposes, mean changes observed in the first year compared to baseline eGFR are shown in the last column.

Supplementary Figure 3 Mean change of eGFR in the 2 years before treatment with tolvaptan compared with the variation observed after treatment with tolvaptan, according to the stage of CKD.

Supplementary Figure 4 Changes in serum uric acid levels in each stage of chronic kidney disease (CKD). Increments of serum uric acid levels were higher in patients with more preserved eGFR, with a slight trend to diminish such increments in more advanced CKD stages. Mean values and mean standard errors are shown.

Supplementary Figure 5 Evolution of prescriptions for uric acid-lowering drugs and chronic kidney disease (CKD) stages. Use of these medications gradually rose as eGFR declined, with a clear increase in prescriptions in CKD2 and CKD3 stages.

Supplementary Figure 6 **(A)** Evolution of use of antihypertensive drugs and diuretics during the first year of treatment with tolvaptan. **(B)** Evolution of the number of antihypertensive drugs during the first year of treatment with tolvaptan.
