# Supplementary material for: Real clinical experience after one year of treatment with tolvaptan in patients with autosomal dominant polycystic kidney disease
Source: Front Med (Lausanne). 2022 Sep 29;9:987092. doi: 10.3389/fmed.2022.987092 (PMC9557750; doi:10.3389/fmed.2022.987092)
Supplement: Supplementary file 1 [file Table_1.DOCX]

Supplementary Table 1. Evolution of the estimated glomerular filtration rate in patients after treatment with tolvaptan according to the Mayo Classification and genetic study.

|  | Baseline | Month 1 | Month 3 | Month 6 | Month 12 |
| --- | --- | --- | --- | --- | --- |
| Mayo Classification |  |  |  |  |  |
| 1C (N= 23):  GFR (mL/min/1.73 m^2^)  Δ from baseline  Δ from first month  1D (N= 54):  GFR (mL/min/1.73 m^2^)  Δ from baseline  Δ from first month  1E (N= 33):  GFR (mL/min/1.73 m^2^)  Δ from baseline  Δ from first month | 55.2±17.4  66.5±23.2  63.0±33.7 | 49.9±15.1 ^a^  -5.3±8.8 ^a^  61.3±24.6 ^b^  -5.2±8.7 ^b^  57.3±29.6 ^c^  -5.7±9.0 ^c^ | 51.3±19.0 ^a^  -4.0±7.4 ^a^  -1.4±9.0  63.2±24.6 ^c^  -4.3±8.2 ^c^  0.4±7.2  57.8±30.0 ^a^  -3.2±9.6 ^a^  0.5±5.8 | 52.1±18.6 ^a^  -3.6±7.2 ^a^  -1.8±10.0  62.2±24.7 ^b^  -4.9±8.5 ^b^  0.1±6.4  57.8±28.2 ^c^  -6.1±9.8 ^c^  -0.2±7.5 | 50.0±19.5 ^a^  -5.2±7.5 ^a^  -0.1±9.0  61.8±24.3 ^b^  -5.9±9.4 ^b^  -0.7±7.8  54.4±27.0 ^b^  -9.5±10.5 ^b^  -3.3±7.3 |
| *PKD1* mutation |  |  |  |  |  |
| Truncating (n= 47)  Not truncating (n=44) | 65.6±27.8  65.8±29.0 | 61.1±26.7 ^a^  61.9±28.5 ^a^ | 61.7±27.1 ^a^  61.7±28.6 ^a^ | 61.2±26.2 ^c^  60.6±28.7 ^b^ | 61.1±26.3 ^a^  58.5±29.1 ^b^ |
| Δ = eGFR change from eGFR at baseline and from first month.  ^a^ p<0.05; ^b^ p<0.001; ^c^ p=0.001; | | | | | |
