# Supplementary material for: Real clinical experience after one year of treatment with tolvaptan in patients with autosomal dominant polycystic kidney disease
Source: Front Med (Lausanne). 2022 Sep 29;9:987092. doi: 10.3389/fmed.2022.987092 (PMC9557750; doi:10.3389/fmed.2022.987092)
Supplement: Supplementary file 2 [file Table_2.DOCX]

Supplementary Table 2. Evolution of estimated glomerular filtration rate (eGFR), serum sodium and uric acid levels and morning urinary osmolality in patients excluded excluded of the study while on treatment with tolvaptan. Number of patients decreased progressively and not all contributed at all times in comparisons of eGFR, so that is difficult to show a clear trend.

|  | Baseline | Month 1 | Month 3 | Month 6 | Month 12 |
| --- | --- | --- | --- | --- | --- |
| N | 57 | 44 | 36 | 32 | 17 |
| eGFR (mL/min/1.73 m^2^)  Δ from baseline  Δ from first month | 58.7±30.1 | 56.3±31 ^a^  -2.3±8.0 | 49.6±25.7 ^b^  -5.1±7.7  -2.6±6.9 | 51.8±30.5 ^a^  -3.8±8.7  -2.6±8.3 | 59.5±36.5  -2.6±10.7  -2.6±8.1 |
| Serum sodium (mEq/L) | 140.3±2.6 | 141.1±3.1 ^a^ | 141.6±2.2 | 140.7±1.9 | 140.2±2.9 |
| Serum uric acid (mg/dL) | 6.1±1.7 | 6.3±1.6 ^a^ | 6.4±1.7 | 6.4±1.4 | 6.4±1.4 |
| Morning urinary osmolality (mOsm/kg) | 460±104 | 252±76 ^a^ | 200±69 ^b^ | 219±63 ^b^ | 207±64 ^c^ |
| Δ = eGFR change from eGFR at baseline and from first month.  ^a^ p<0.001; ^b^ p=0.001; ^c^ p<0.05 | | | | | |
