# Supplementary figures and images for: Real clinical experience after one year of treatment with tolvaptan in patients with autosomal dominant polycystic kidney disease
Source: Front Med (Lausanne). 2022 Sep 29;9:987092. doi: 10.3389/fmed.2022.987092 (PMC9557750; doi:10.3389/fmed.2022.987092)

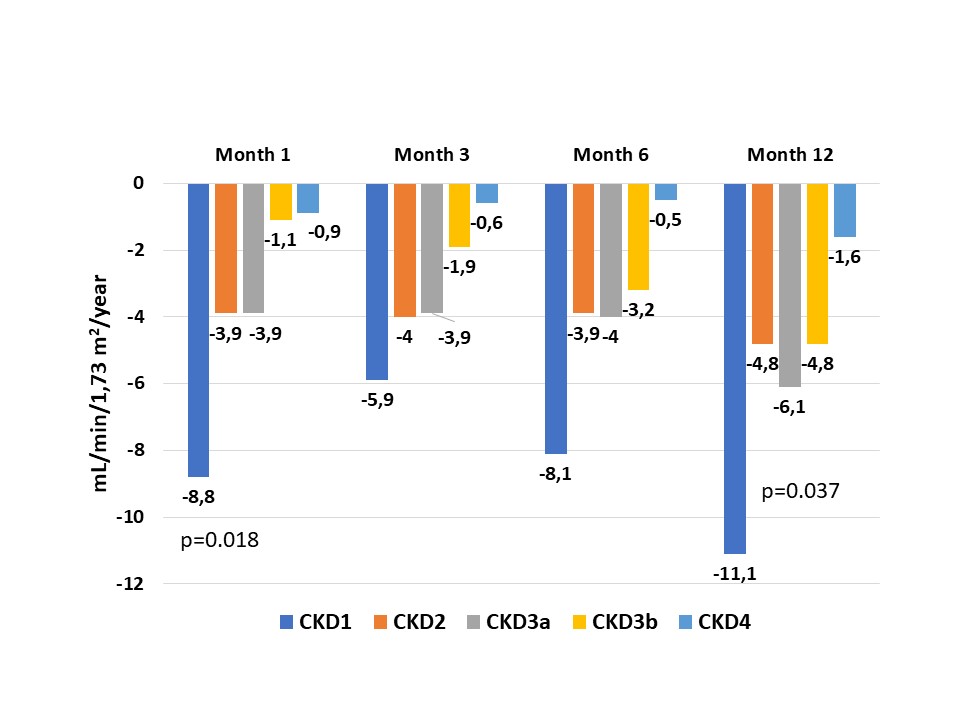

Supplement: Supplementary Figure 1 — Changes in eGFR compared to its baseline value, according to the stage of CKD, during the first year of treatment with tolvaptan. A progressive decrease in eGFR was observed from the first month of treatment in all stages except stage CKD4. The most significant decrease was in the CKD1 stage. Mean values and mean standard errors are shown. [file Image_1.JPEG]

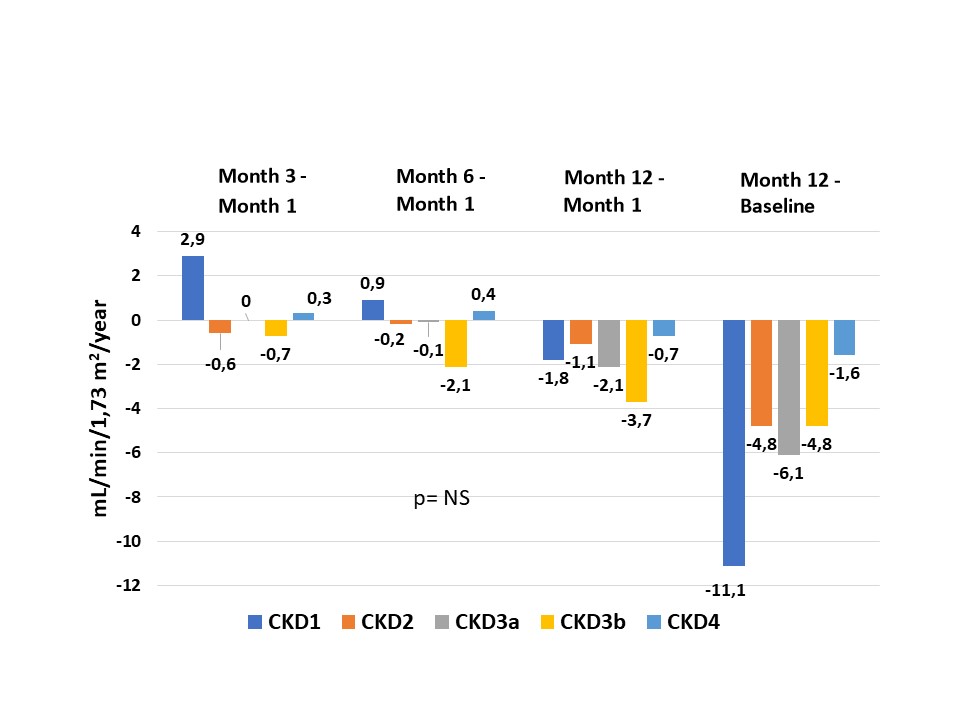

Supplement: Supplementary Figure 2 — Mean difference of the eGFR compared to its value in the first month of treatment with tolvaptan. Changes observed were not significant in any stage of CKD. For comparison purposes, mean changes observed in the first year compared to baseline eGFR are shown in the last column. [file Image_2.JPEG]

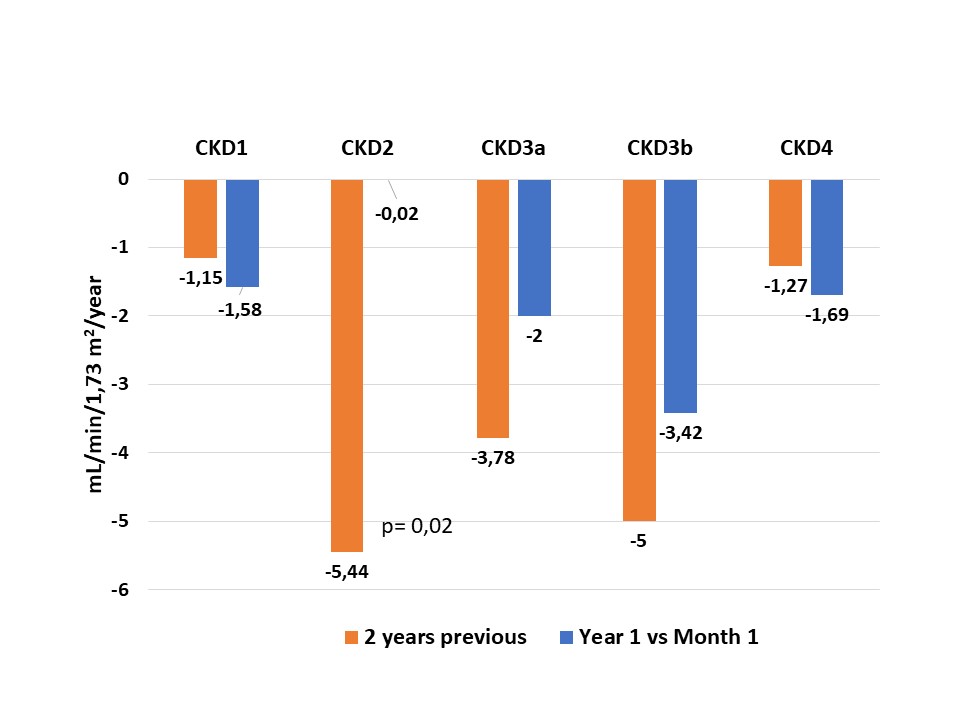

Supplement: Supplementary Figure 3 — Mean change of eGFR in the 2 years before treatment with tolvaptan compared with the variation observed after treatment with tolvaptan, according to the stage of CKD. [file Image_3.JPEG]

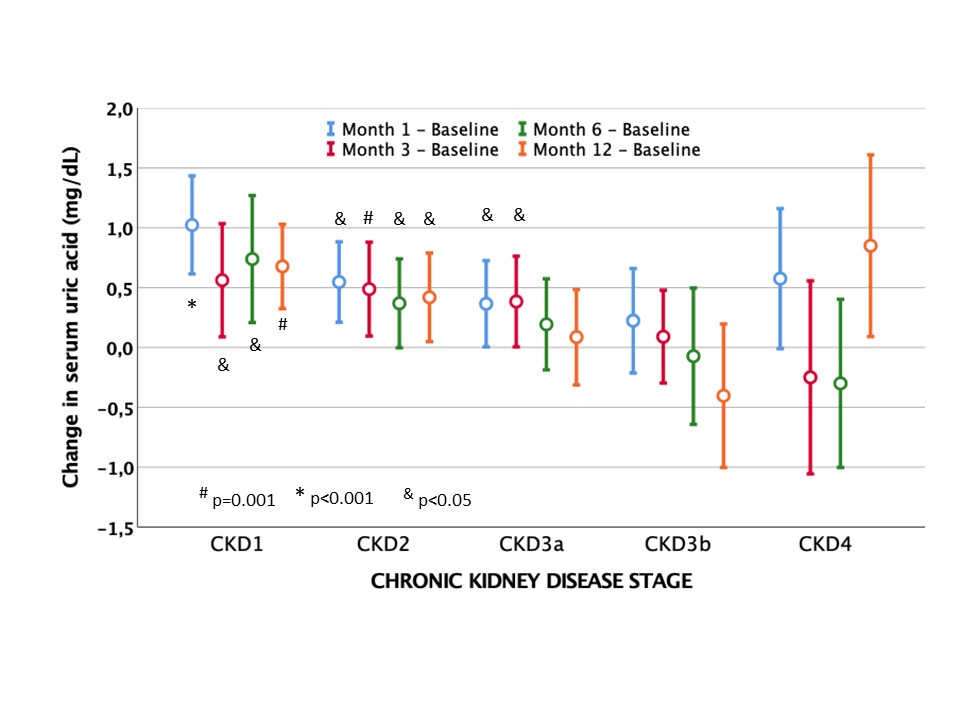

Supplement: Supplementary Figure 4 — Changes in serum uric acid levels in each stage of chronic kidney disease (CKD). Increments of serum uric acid levels were higher in patients with more preserved eGFR, with a slight trend to diminish such increments in more advanced CKD stages. Mean values and mean standard errors are shown. [file Image_4.JPEG]

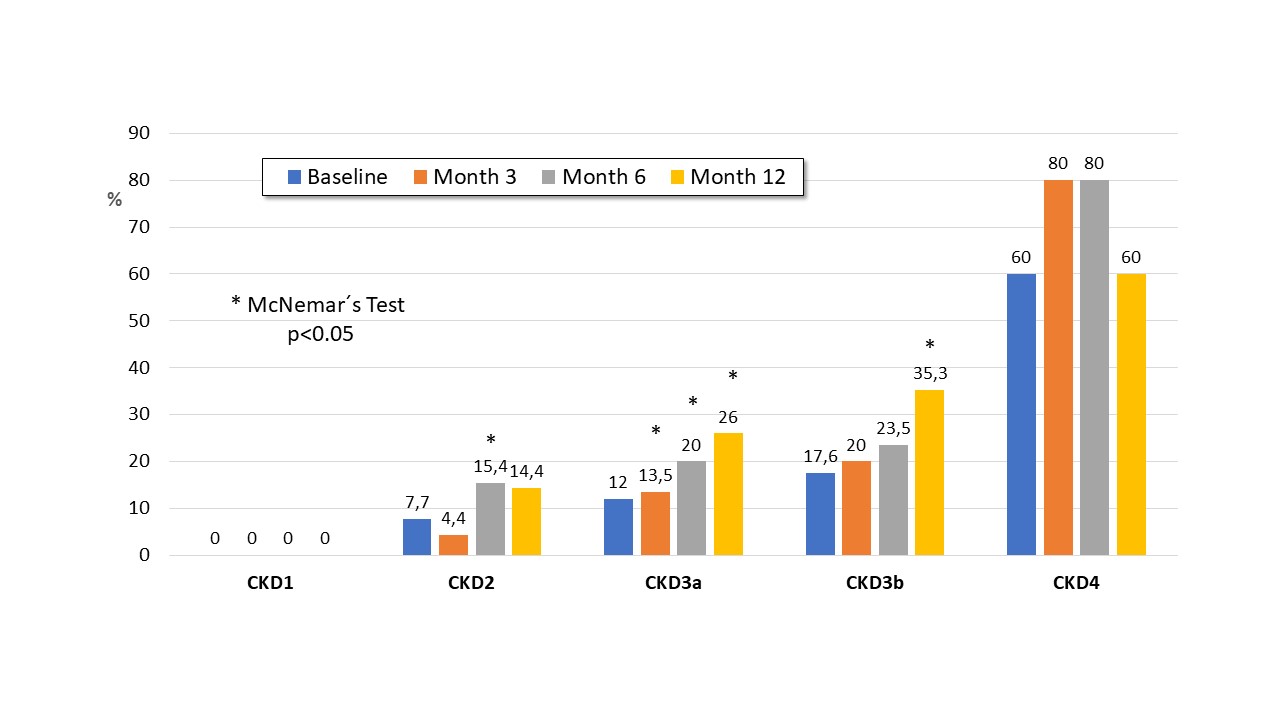

Supplement: Supplementary Figure 5 — Evolution of prescriptions for uric acid-lowering drugs and chronic kidney disease (CKD) stages. Use of these medications gradually rose as eGFR declined, with a clear increase in prescriptions in CKD2 and CKD3 stages. [file Image_5.JPEG]

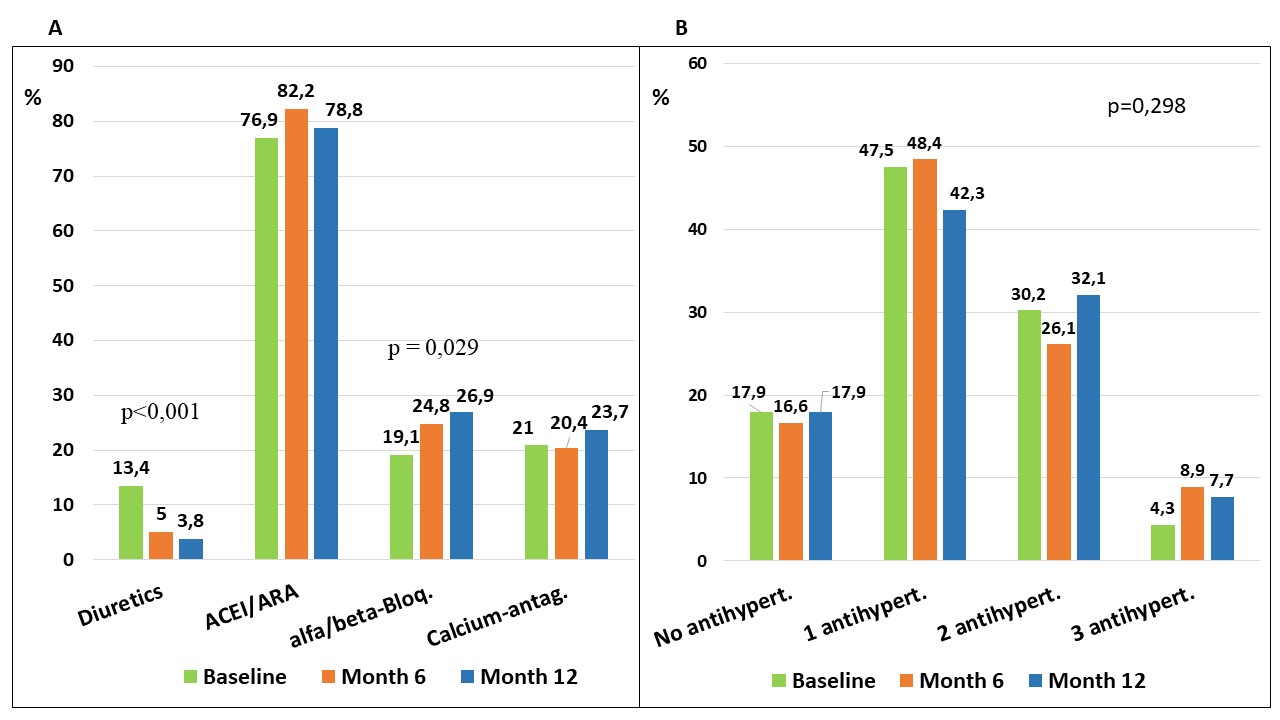

Supplement: Supplementary Figure 6 — (A) Evolution of use of antihypertensive drugs and diuretics during the first year of treatment with tolvaptan. (B) Evolution of the number of antihypertensive drugs during the first year of treatment with tolvaptan. [file Image_6.JPEG]
